# Complementary utility of targeted next-generation sequencing and immunohistochemistry panels as a screening platform to select targeted therapy for advanced gastric cancer

**DOI:** 10.18632/oncotarget.16409

**Published:** 2017-03-21

**Authors:** Hyo Song Kim, Hanna Lee, Su-Jin Shin, Seung-Hoon Beom, Minkyu Jung, Sujin Bae, Eun Young Lee, Kyu Hyun Park, Yoon Young Choi, Taeil Son, Hyoung-Il Kim, Jae-Ho Cheong, Woo Jin Hyung, Jun Chul Park, Sung Kwan Shin, Sang Kil Lee, Yong Chan Lee, Woong Sub Koom, Joon Seok Lim, Hyun Cheol Chung, Sung Hoon Noh, Sun Young Rha, Hyunki Kim, Soonmyung Paik

**Affiliations:** ^1^ Division of Medical Oncology, Department of Internal Medicine, Yonsei University College of Medicine, Seoul, Republic of Korea; ^2^ Severance Biomedical Science Institute, Yonsei University College of Medicine, Seoul, Republic of Korea; ^3^ Hanyang University, College of Medicine, Seoul, Republic of Korea; ^4^ Cancer Metastasis Research Center, Song Dang Institute for Cancer Research, Yonsei University College of Medicine, Seoul, Republic of Korea; ^5^ Department of Surgery, Yonsei University College of Medicine, Seoul, Republic of Korea; ^6^ Division of Gastroenterology, Department of Internal Medicine, Yonsei University College of Medicine, Seoul, Republic of Korea; ^7^ Department of Radiation Oncology, Yonsei University College of Medicine, Seoul, Republic of Korea; ^8^ Department of Radiology, Yonsei University College of Medicine, Seoul, Republic of Korea

**Keywords:** gastric cancer, molecular subtypes, next-generation sequencing, immunohistochemistry, matched therapy

## Abstract

We tested the clinical utility of combined profiling of Ion Torrent PGM based next-generation sequencing (NGS) and immunohistochemistry (IHC) for assignment to molecularly targeted therapies. A consecutive cohort of 93 patients with advanced/metastatic GC who underwent palliative chemotherapy between March and December 2015 were prospectively enrolled. Formalin fixed paraffin embedded tumor biopsy specimens were subjected to a 10 GC panels [Epstein Barr virus encoding RNA *in-situ* hybridization, IHC for mismatch repair proteins (MMR; MLH1, PMS2, MSH2, and MSH6), receptor tyrosine kinases (HER2, EGFR, and MET), PTEN, and p53 protein], and a commercial targeted NGS panel of 52 genes (Oncomine Focus Assay). Treatment was based on availability of targeted agents at the time of molecular diagnosis. Among the 81 cases with available tumor samples, complete NGS and IHC profiles were successfully achieved in 66 cases (81.5%); only IHC results were available for 15 cases. Eight cases received matched therapy based on sequencing results; *ERBB2* amplification, trastuzumab (*n* = 4); *PIK3CA* mutation, Akt inhibitor (*n* = 2); and *FGFR2* amplification, FGFR2b inhibitor (*n* = 2). Eleven cases received matched therapy based on IHC; *ERBB2* positivity, trastuzumab (*n* = 5); PTEN loss (*n* = 2), PI3Kβ inhibitor; MMR deficiency (*n* = 2), PD-1 inhibitor; and EGFR positivity (*n* = 2), pan-ERBB inhibitor. A total of 19 (23.5%) and 62 (76.5%) cases were treated with matched and non-matched therapy, respectively. Matched therapy had significantly higher overall response rate than non-matched therapy (55.6% vs 13.1%, *P* = 0.001). NGS and IHC markers provide complementary utility in identifying patients who may benefit from targeted therapies.

## INTRODUCTION

Despite combined treatment with surgical resection and adjuvant chemotherapy, 25–40% of patients with stage II-IV Gastric cancer (GC) experience relapse [1, 2]. Large-scale molecular profiling of GC, as reported in The Cancer Genome Atlas (TCGA) and Asian Cancer Research Group (ACRG), identified multiple cancer drivers as potential therapeutic targets [3, 4]. However, chemotherapy remains the only treatment option for patients diagnosed with advanced GCs with dismal outcome, with the exception of trastuzumab for a HER2-positive GC subset, based on the results of the TOGA trial [5].

Advances in genome sequencing technology have allowed the identification of potential therapeutic targets using formalin fixed paraffin embedded (FFPE) tumor biopsy specimens within a timeframe compatible with clinical practice [6]. Using a genome forward designs, clinical trials have been conducted to investigate the efficacy of targeted agents against specific molecular aberrations in a single or multiple tumor types [7–11]. Findings from histology-agonistic approaches demonstrated improved progression free survival (PFS) and overall survival (OS) compared to those achieved with non-matched therapy [11, 12]. However, in randomized trial with heavily treated solid tumors, targeted agents did not improve the PFS over physician's choice [9]. As a histology-dependent approach, BATTLE and SAFIRO01 trials established the feasibility of a genome forward approach in lung and breast cancer patients [7, 13].

Regarding GC, despite recent studies with comprehensive molecular profiling [3, 4, 14, 15], no clinical data demonstrating target-drug efficacy in the context of umbrella studies have been published yet. In addition, the very small sizes of FFPE gastric biopsy specimens pose practical and technical challenges often result in sequencing assay failures due to low yield and poor quality of extracted DNA. Therefore, we combined Ion Torrent PGM based amplicon sequencing (Oncomine Focus Assay, Thermo Fisher Scientific, Waltham, MA, USA) with an immunohistochemistry (IHC) panel to maximize the chance of assignment of enrolled patients to potentially beneficial targeted therapies. We also aimed to assess whether our genome forward umbrella approach could improve patient outcomes when compared to non-matched, standard chemotherapy for advanced/metastatic GC.

## RESULTS

### Patients and molecular aberrations

#### Sample set and clinicopathologic characteristics

Among the total 81 patients, 50 (61.7%) were male with the median age of 57 years (range 28–76). Biopsy samples (50 endoscopic biopsies and 8 excisional biopsies for metastatic sites) were used for two-thirds of cases (*n* = 58, 71.6%). Nineteen patients (23.5%) were treated with matched therapy and 62 (76.5%) were treated with non-matched therapy (Table 1).

**Table 1:** Clinicopathological characteristics

Characteristics	No.	%	Matched (%)	Non-matched (%)	*P*
**Total**	81		19 (23.5%)	62 (76.5%)	
**Age, years**					
Median (range)	57 (28–76)		59 (29–73)	57 (28–76)	0.95
**Gender**					
Male	50	61.7	12 (63.2%)	38 (61.3%)	0.88
Female	31	38.3	7 (36.8%)	24 (38.7%)	
**Differentiation**					
Well	1	1.2	1 (5.3%)	0	0.20
Moderate	24	29.6	8 (42.1%)	16 (25.8%)	
Poorly	38	46.9	7 (36.8%)	31 (50.0%)	
Signet ring cell	16	19.8	3 (15.8%)	13 (21.0%)	
Others	2	2.5	0	2 (3.2%)	
**Tumor location**					
Upper	8	9.9	1 (5.3%)	7 (11.3%)	0.87
Body	27	33.3	6 (31.6%)	21 (33.9%)	
Antrum	38	46.9	10 (52.6%)	28 (45.2%)	
Entire	8	9.9	2 (10.5%)	6 (9.7%)	
**Tissue samples**					
Gastrectomy	23	28.4	2 (10.5%)	21 (33.9%)	0.14
Endoscopic biopsy	50	61.7	15 (78.9%)	35 (56.5%)	
Biopsy formetastatic sites	8	9.9	2 (10.5%)	6 (9.7%)	
**Stage at diagnosis**					
I	3	3.7	1 (5.3%)	2 (3.2%)	0.55
II	7	8.6	1 (5.3%)	6 (9.7%)	
III	11	13.6	1 (5.3%)	10 (16.1%)	
IV	60	74.1	16 (84.2%)	44 (71.0%)	
**Metastatic site**					
Peritoneum	45	55.6	9 (47.4%)	36 (58.1%)	0.41
Lymph node	26	32.1	9 (47.4%)	17 (27.4%)	0.06
Liver	23	28.4	9 (47.4%)	14 (22.6%)	0.05
Lung	5	6.2	2 (10.5%)	3 (4.8%)	0.37
Bone	2	2.5	1 (5.3%)	2 (3.2%)	0.87
**Prior therapies**					
Treatment naive	33	40.7	9 (47.4%)	24 (38.7%)	0.50
1–2	48	59.3	10 (52.6%)	38 (61.3%)	

#### Molecular profiling: mutation, amplification, and overexpression

For all 81 cases, we profiled 10 molecular markers using IHC and *in situ* hybridization (ISH). From a combination of markers, we observed a median of 1 genomic aberration per case (range 0–4). Of those, Epstein-Barr virus (EBV) was identified by ISH in 3 (3.7%) case, and 2 of those harbored the *PIK3CA* Q546K mutation upon NGS. Mismatch repair (MMR) deficiency was demonstrated in 5 (6.2%) cases, all of which exhibited simultaneous losses of MLH1 and PMS2 expression. Receptor tyrosine kinase (RTK) overexpression occurred in 46 (56.8%) cases, when scores of 2+ and 3+ were defined as positive expression. [16] PTEN loss was observed in 15 patients (18.5%).

**Figure 1:**
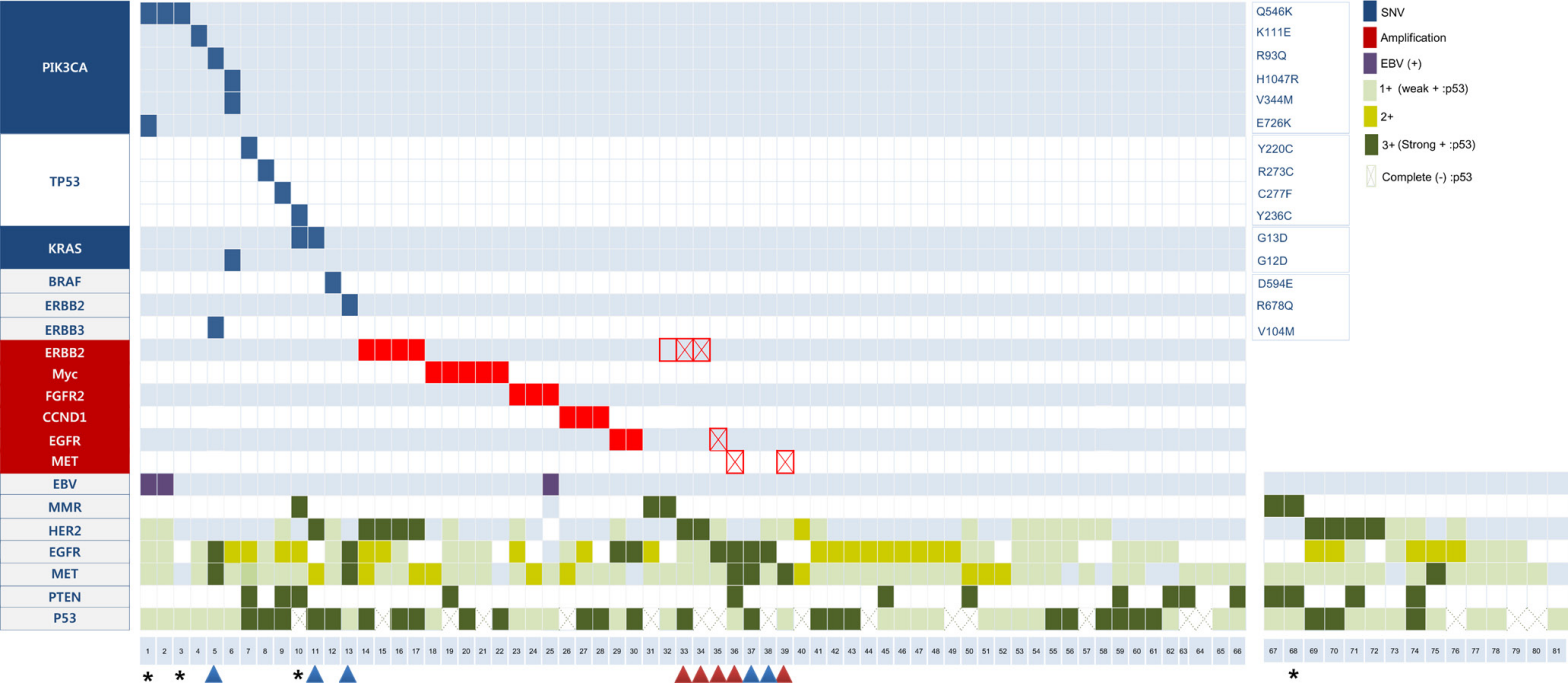
Mutation, amplification, and protein expression profiles Vertical lines indicate gene names; horizontal lines indicate the cases. Red and blue arrows indicate cases with concordant IHC and NGS, respectively. Empty squares indicate false-positive *ERBB2* amplification on NGS. Squares with diagonal lines indicate false-negative RTK amplification on NGS. Asterisks denote representative cases treated with matched therapy.

Complete NGS and IHC profiles were available for 66 cases (81.5%). At least 1 mutation or amplification was found in 30 (45.5%), of which 26 (86.7%) harbored single aberrations and 4 (13.3%) had multiple aberrations (Figure 1). The most common mutations were as follows; *PIK3CA* (*n* = 6, 9.1%) *TP53* [*n* = 4, 40% among 10 cases with Oncomine Comprehensive Assay (OCA)], and *KRAS* (*n* = 3, 4.5%). All *PIK3CA* mutations, including Q546K (*n* = 3), K111E (*n* = 1), R93Q (*n* = 1), H1047R (*n* = 1), V344M (*n* = 1), and 3 were co-occurrence mutations with *KRAS* (G12D), *PIK3CA (E726K)*, and *ERBB3* (V104M). Non-V600E *BRAF* mutation (D594E) was detected in 1 case.

Amplification was detected in 17 cases (25.8%) and was mutually exclusive with mutation. Among those, 16 cases (94.1%) exhibiting amplification on NGS also exhibited gene amplification on SISH or FISH assay (Appendix Table 2 and Appendix Figure 2A–2E). One case exhibited *ERBB2* amplification on NGS but neither SISH amplification nor HER2 expression RTK (#32 in Figure 1). Among the 10 cases that exhibited receptor tyrosine kinase (RTK) overexpression via IHC (3+) in the absence of corresponding gene amplification (Appendix Table 3), 5 cases exhibited *ERBB2* (*n* = 2), *MET* (*n* = 2), or *EGFR* (*n* = 1) amplification by SISH concordant with the IHC results (Appendix Figure 2F–2G, red arrows in Figure 1). However, the other 5 cases did not exhibit gene amplification in agreement with the NGS results (Appendix Figure 2H–2I, blue arrows in Figure 1).

### Treatment assignment and clinical outcomes

#### Patients with or without molecular aberrations

**Figure 2:**
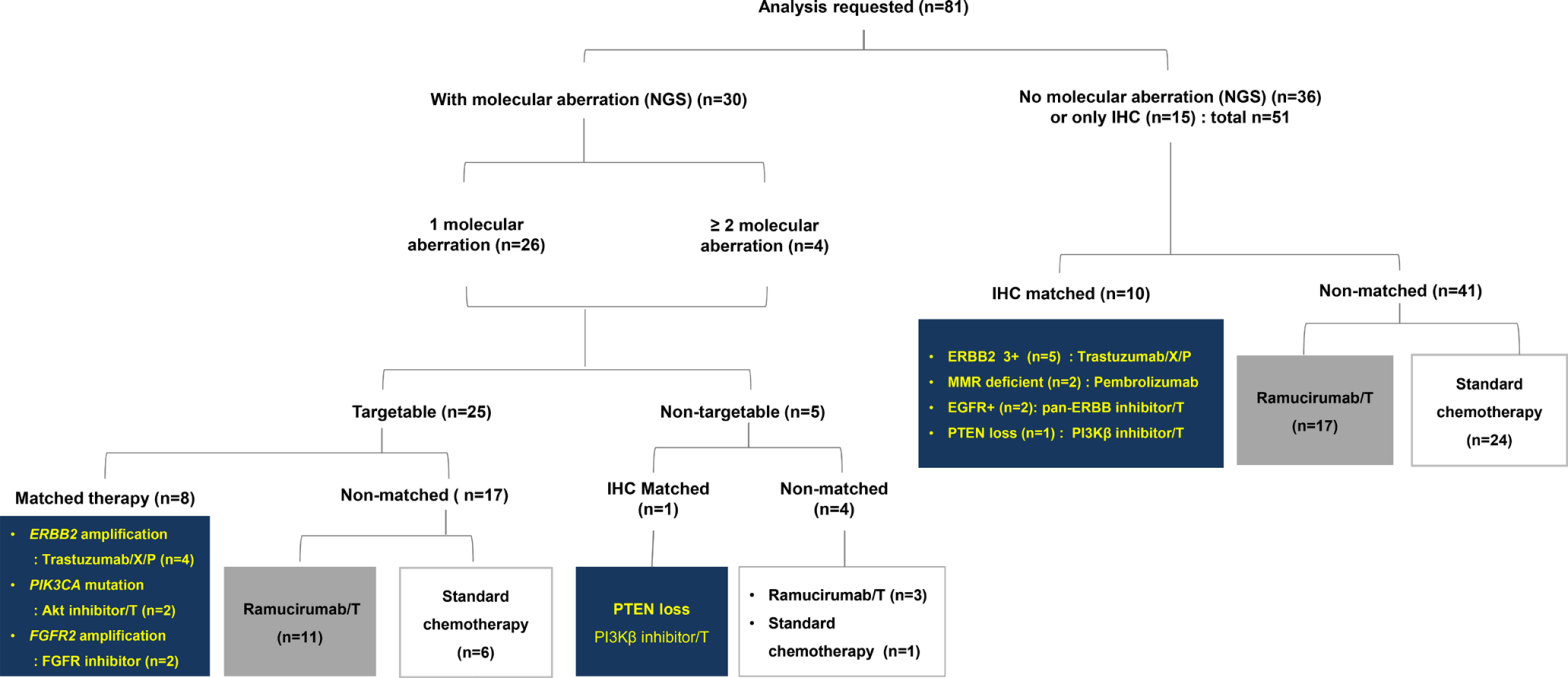
CONSORT diagram T: paclitaxel, X: capecitabine, C: cisplatin.

Of the 30 cases with NGS-detected genetic aberrations, 9 were treated with matched therapy and 21 were treated with non-matched therapy (Figure 2). Among those matched therapy, *ERBB2* amplification (*n* = 4) were treated with trastuzumab-containing chemotherapy (trastuzumab, capecitabine, and cisplatin), and *PIK3CA* mutation (*n* = 2) were treated with oral Akt inhibitor, afuresertib in combined with paclitaxel (ClinicalTrials.gov Identifier: NCT02240212). *FGFR2* amplification (*n* = 2) were treated with FPA144 monotherapy, antibody to *FGFR2b* receptor, (ClinicalTrials.gov Identifier: NCT02318329), and PTEN loss (*n* = 1) received matched therapy with GSK2636771, PI3Kβ inhibitor in combined with paclitaxel (ClinicalTrials.gov Identifier: NCT02615730).

For the 51 remaining cases, 10 were treated with matched therapy according to the IHC results; trastuzumab containing chemotherapy for ERBB2 positivity (3+, *n* = 5), PI3Kβ inhibitor for PTEN loss (*n* = 1), pan-ERBB tyrosine kinase inhibitor, afatinib with paclitaxel (ClinicalTrials.gov Identifier: NCT02501603) for EGFR positivity (*n* = 2), and pembrolizumab-containing regimen, a monoclonal antibody to programmed cell death 1 (PD-1) inhibitor for MMR-deficiency (*n* = 2).

#### Responses and survival

**Figure 3:**
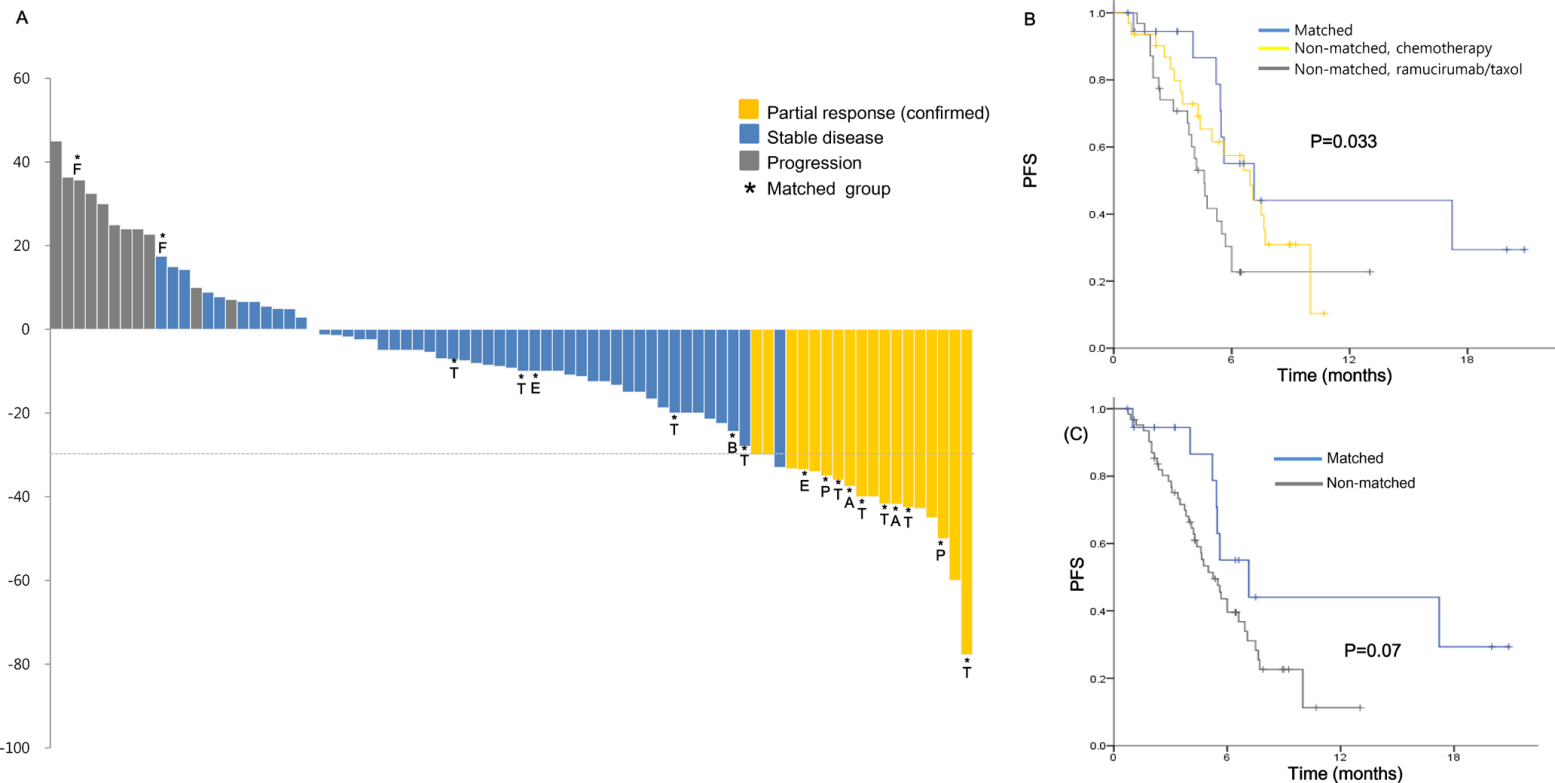
Efficacy data based on molecular profiling (**A**) Waterfall plot of all patients demonstrating the maximum percent change with respect to baseline. Progression-free survival (**B**) and overall survival (**C**). T; trastuzumab, P; pembrolizumab, A; Akt inhibitor, B; PI3Kβ inhibitor, E; pan-ERBB inhibitor.

Of the 79 evaluable patients, 18 patients (22.8%) had confirmed partial responses (cPR) and 50 (63.3%) had stable diseases (Appendix Table 4). Patients who received matched therapy had a higher overall response rate (ORR) of 55.6%, compared with 13.1% for those treated with non-matched therapy (Figure 3A, *P* = 0.001). With median 19.6 months of follow-up, the median PFS were 7.1 months for matched therapy [95% confidence interval (CI), 3.0–11.2], 4.6 months for non-matched ramucirumab/paclitaxel group (95% CI, 3.8–5.4), and 6.9 months for non-matched chemotherapy group (95% CI, 4.8–9.0), respectively (*P* = 0.033, Figure 3B). The median PFS was slightly longer for matched group compared to non-matched group (7.1 *vs* 5.2 months, *P* = 0.07).

#### Clinical response to matched therapy

**Figure 4:**
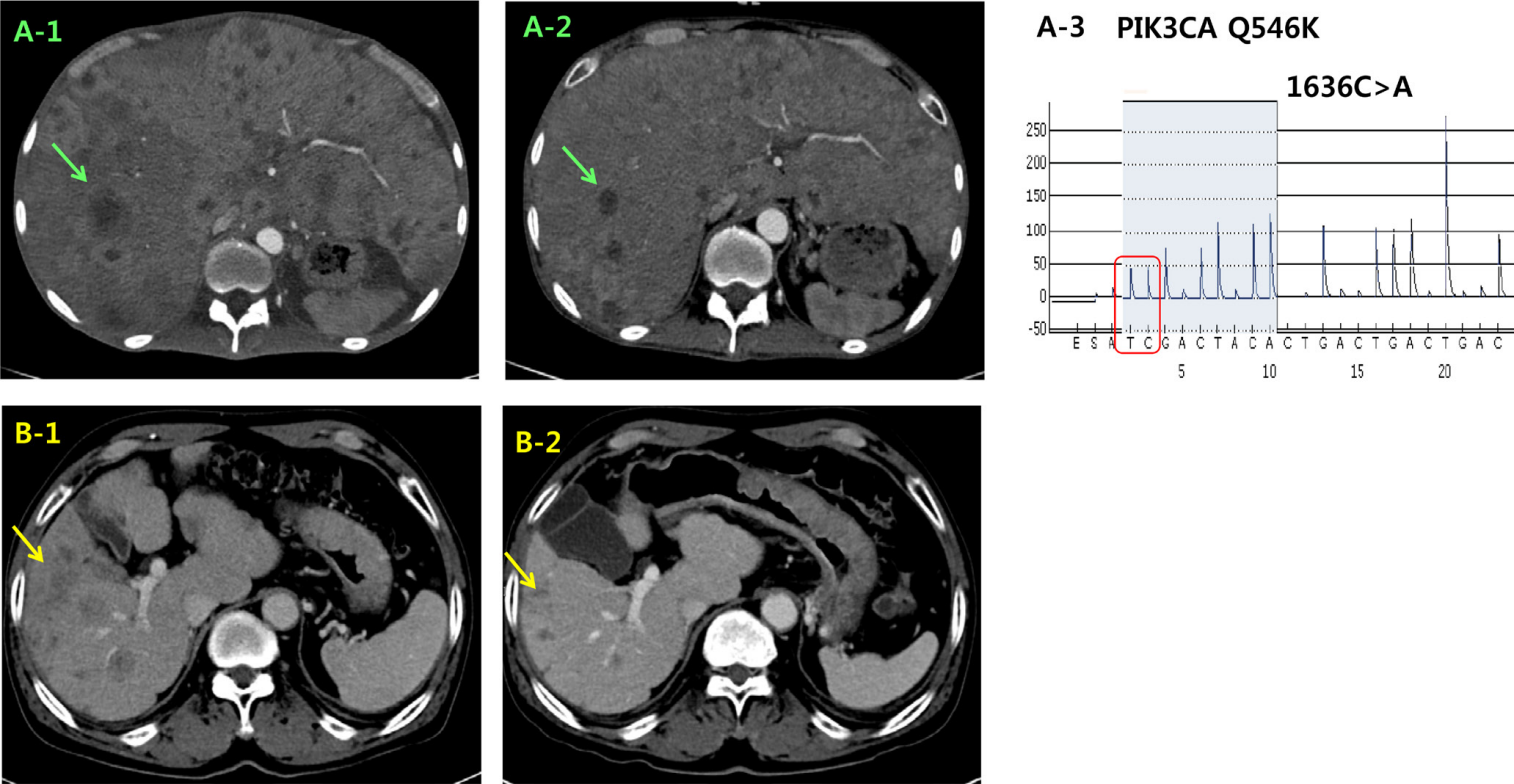
Representative clinical responses of 2 *PIK3CA* mutant cases in the matched group that were treated with the combination of an Akt inhibitor and paclitaxel CT images of case #3 during the treatment course. The initial liver metastasis (**A1**) exhibited a significant size reduction (**A2**) after 12 weeks. (**A3**) *PIK3CA* exon 9 Q546K mutation was detected by pyrosequencing. CT images of case #1 at the time of baseline (**B1**) and at 16 weeks (**B2**). CT: computed tomography.

Two patients with *PIK3CA* Q546K mutation were enrolled in a phase IB clinical trial with Akt inhibitor. The first GC patient (Case #3 in Figure 1) presented with hepatic metastases was treated with 200 mg of daily afuresertib combined with weekly paclitaxel as second line treatment. A computed tomography (CT) scan at the end of cycle 3 (12 weeks) showed cPR to treatment with a 41.8 % reduction (Figure 4A). The patient was removed from the study with a time to progression of 25 weeks. Second case (Case #1 in Figure 1) was 67-year-old male with paraaortic and hepatic metastases. He was treated with afuresertib and paclitaxel. A CT scan at 16 weeks demonstrated cPR with a 37.7% tumor reduction (Figure 4B), and he remains on treatment after 17 weeks.

**Figure 5:**
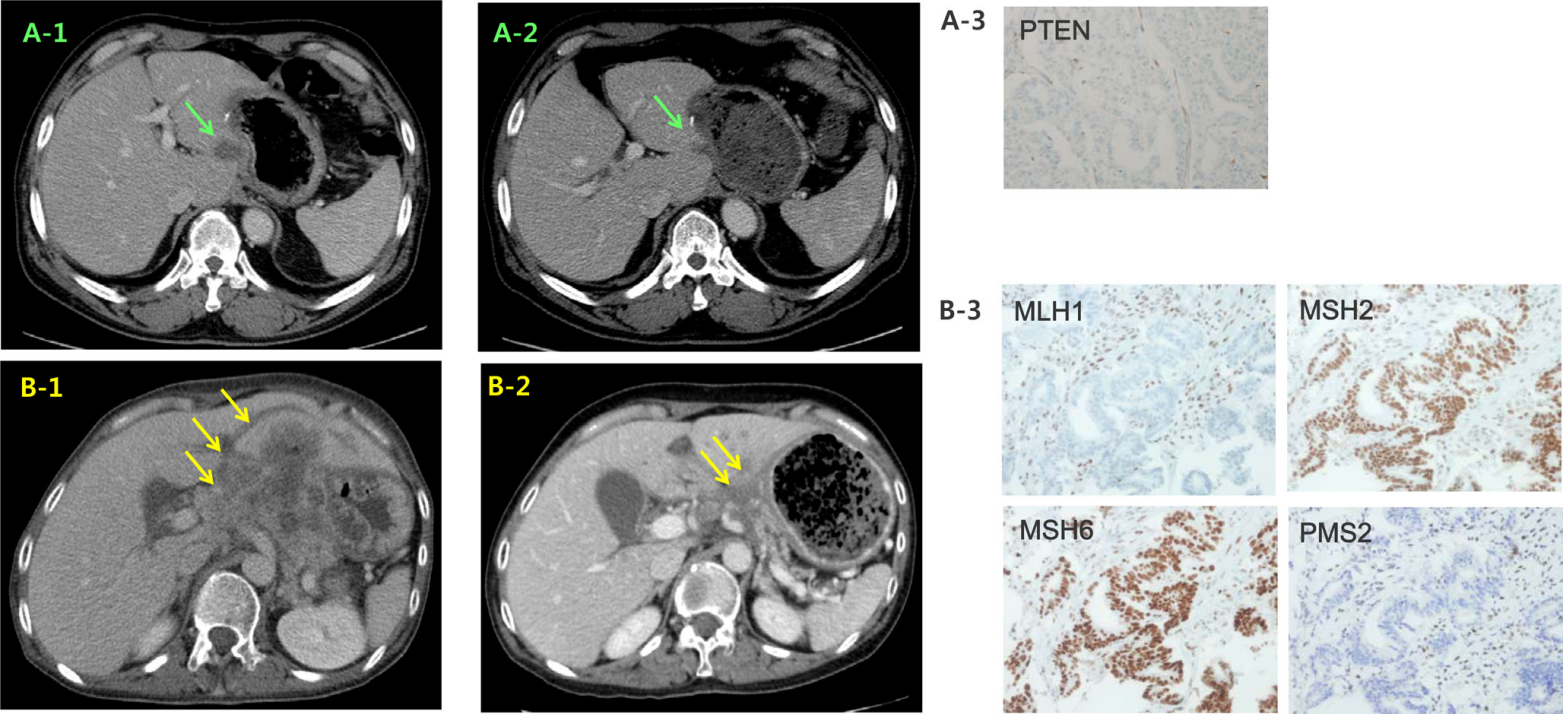
Representative clinical responses in the matched group CT images of case #10 at baseline (**A1**) and 12 weeks (**A2**) after treatment with a PI3Kβ inhibitor and paclitaxel. (**B**) CT images of case #68 at baseline and 12 weeks (**B2**) after pembrolizumab treatment. Immunohistochemistry images indicating PTEN loss in case #10 (**A3**) and MMR deficiency in #68 (**B3**).

The third case (Case #10 in Figure 1) developed multiple hepatic recurrences 7 months after curative resection (initially stage IB). The tumor was PTEN loss (with H-score 60 out of 300) and he was treated with 300 mg of daily GSK2636771 in combined with weekly paclitaxel. A CT scan at 12 weeks demonstrated stable disease with a 24% tumor reduction (Figure 5A) and he remains on treatment after 25 weeks.

A 53-year-old female patient with a MMR deficiency (Case #68 in Figure 1) was also treated with the PD-1 inhibitor, pembrolizumab (200 mg) every 3 weeks combination with TS-1 monotherapy (50 mg bid, days 1–14). A CT scan at 12 weeks indicated cPR with a 49.8% tumor reduction; she is on treatment after 34 weeks (Figure 5B).

## DISCUSSION

In recognition of inter-patient molecular tumor heterogeneity, NGS-based basket trials with specific molecular aberrations across multiple tumor types were widely studied [9–11]. However, in SHIVA trial, the off-label use of molecularly targeted agents did not improve PFS in heavily pretreated cancer patients when compared with physician's choice [9]. As demonstrated by the different efficacy of BRAF inhibitors in melanoma and colon cancer [17], the inclusion of multiple tumor types with the same molecular target in a basket trial might introduce an important source of heterogeneity and could lead to negative results.

These observations justify a shift toward a histology dependent approach involving targeted agents. The BATTLE study demonstrated the feasibility and utility of umbrella approach for advanced non-small cell lung cancer (NSCLC) [13]. TCGA classified GCs into four molecular subtypes; EBV-positive tumors (8.8%), microsatellite instability-high (MSI-H) tumors (21.6%), genomically stable tumors (GS, 19.6%), and tumors with chromosomal instability (CIN, 49.6%) [3]. In our study, combination of OFA and IHC panels covered most of the key druggable targets for each subgroup. Our pilot study results demonstrated that the matched group experienced significantly better responses and survival and provides justifying the need for further umbrella trials in GC. We await the results of two ongoing umbrella trials PANGEA and VIKTORY We await the results of 3 ongoing umbrella trials for GC(ClinicalTrials.gov Identifier: NCT02213289, NCT02299648 and NCT02951091). (ClinicalTrials.gov Identifier: NCT02213289 and NCT02299648).

Genomic profiling of biopsied metastatic lesions often presents practical challenges because of the small quantities of available samples. The OCA, which is based on Ion Torrent PGM amplicon sequencing platform is currently used as a screening platform for the NCI MATCH study because of its low sample requirement (20 ng DNA and 10ng RNA from FFPE specimens), and ability to detect CNAs and gene fusions as well as SNVs. The OFA is a subset of OCA that mainly targets actionable cancer drivers. The success rate of our genomic analyses (71%, 66 /93) was similar to those reported trials that used large-scale genomic analyses, with main reason of failure being low cellularity and DNA contents [7, 9, 12].

Despite the known difficulty of reliable CNA detection from amplicon-based targeted sequencing data (because of variable amplification efficiency across targets) [14, 18], OFA with a proprietary analysis pipeline with *in silico* reference normal tissue data could identify 17 CNAs, 16 (94.1%) of which were validated by ISH. However, the IHC panel was able to identify an additional 5 *ERBB2* amplified cases, which were confirmed by ISH. OFA alone would have missed 23% of the CNAs. On the other hand, the additional 5 candidate CNAs identified by the IHC panel (no NGS amplification, 3+ RTK overexpression by the IHC panel) were not detected by ISH or NGS. Overall, more than a half (*n* = 11) of the decisions regarding matched therapy were made based on IHC, underscoring the importance of combining NGS with an IHC panel. By applying a more sophisticated approach that incorporated both IHC and NGS, our cancer profiling led to the use of matching therapy in a greater proportion of cases (23.5%), as well as a higher ORR than with previous studies [7, 9].

There are some important issues must be considered when interpreting of this study. First, this was not a randomized study, and therefore results must be interpreted cautiously. However, PFS of the non-matched (control) group in our study was consistent with those reported in phase III randomized trials in support of our results; for example, our study showed a PFS of 4.6 months in the non-matched ramucirumab/paclitaxel group, compared with 4.4 months in the ramucirumab/paclitaxel group from the RAINBOW trial [19]. Second, approximately 40% cases receiving matched therapy is *ERBB2* amplified cases, which is already been identified as a standard biomarker of advanced GC. However, we believe that the inclusion of *ERBB2* amplified cases in our analyses is justified as the primary purpose of the umbrella approach is the identification of all therapeutic targets at the time of diagnosis, thus avoiding the need to successively evaluate single markers over time. Accordingly, *ERBB2* positivity, a main genetic aberration of GC, should be included in this type of study. In support of our approach, two-thirds of matched group was treated according to clinical practice in the NEXT-1 trial [18], and *EGFR*-mutant NSCLCs were also included in the analyses of the BATTLE and CUSTOM trials [10, 13]. Interestingly, one case (case #25) has co-occurrence of EBV positivity and *FGFR2* amplification. Tumor was not responded to FGFR2 inhibitor and efficacy of immune checkpoint inhibitor needs to be determined. Finally, the algorithms used to interpret genomic data and assign targeted therapies need to be improved in the era of immunotherapy and DNA repair modulation.

In this study, we have demonstrated that a combined NGS and IHC analysis of FFPE samples is a feasible method for the identification of targetable genomic alterations in patients with metastatic GC. In addition, identification of specific molecular aberrations and assignment of targeted therapy were associated with better responses and survival supportive for future randomized trial.

## MATERIALS AND METHODS

### Study design and subjects

This study had the following objectives: (1) to test the feasibility of NGS based genomic profiling of small FFPE tumor biopsies routinely produced for diagnostic purpose, in a time frame feasible for clinical practice; (2) to assign patients to matched therapy based on genomic aberrations; and (3) to explore the potential clinical benefit of a genome-forward approach over conventional chemotherapy.

The study scheme is outline in Appendix Figure 1. A consecutive cohort of 93 patients with advanced/metastatic GC who underwent palliative chemotherapy at Severance Hospital, Yonsei University College of Medicine, Seoul, Korea between March 2015 and December 2015 was enrolled in this study. Of those, 12 were excluded from genomic analysis [insufficient tumor cellularity, 11 patients (11.8%); lost to follow-up, 1 patient (1.0%)]. Among the remaining 81 patients, complete NGS and IHC profile were successfully obtained for 66 cases (81.5%). For the remaining 15 cases (18.5%), only IHC data were available because of failed quality control for the NGS analyses (insufficient DNA yield, 8 patients; poor DNA quality, 7 patients). The study was approved by the institutional review board (IRB. 4–2014-0349).

### Biomarker methodology

#### Nucleic acid extraction from FFPE

Ten 4-mm-thick FFPE sections were used for the IHC panel and 2–4 sections were used for NGS. Using hematoxylin and eosin (H&E)-stained sections for guidance, tumor rich areas were reviewed and macrodissected by two experienced pathologists (H.K. and S.J.S) to achieve a final tumor content per sample over 10%. DNA was isolated using the Ambion RecoverAll™ Multi-sample DNA workflow (Thermo Fisher Scientific, Waltham, MA, USA) according to the manufacturer's instructions. DNA was quantified using the Qubit 2.0 fluorometer High sensitivity kit (Thermo Fisher Scientific).

#### Library construction and sequencing

Ten to twenty nanograms of DNA were amplified using Oncomine Comprehensive Assay (OCA, case #1–10 in Figure 1) or Oncomine Focus Assay (OFA, case 11–66) DNA panels targeting 143 and 52 genes, respectively, according to manufacturer's instruction (Appendix Table 1). RNA analysis for fusion transcript was not performed, given the lack of clinically important fusion events in GC.

#### Data analysis

Sequenced data were initially aligned and mapped to the human hg19 reference genome using the Torrent Suite Server (ver 4.4) with default parameters. Amplicon coverage summary files were generated results using the Coverage Analysis plug-in (version 4.4.12–1). The Ion Reporter Workflow (version 5.0) was used to perform variant calling of the DNA libraries. In detail, gene annotation was performed using the Oncomine Panel v1.1 Annotations set and copy number baseline was performed using the Oncomine Panel v2.0. Baseline and Oncomine Variant annotator v2.0 plugin was used for analysis. Analyzed variants were re-categorized using the Oncomine knowledgebase, which includes currently approved drugs and clinical trials. We used the following cutoff values: coverage, > 1000×; and proportion of reads on target, > 80%.

#### Immunohistochemical staining, epstein–barr virus-encoded small RNA-*In Situ* hybridization, and evaluation

IHC was performed on a Ventana XT automated staining instrument (Ventana Medical Systems, Tucson, AZ, USA). The following target-specific antibodies were used according to the manufacturer's instructions and a previous study [20]: MutL homolog 1 (MLH1, ready to use, clone M1, Roche, Basel, Switzerland), MutS protein homolog 2 (MSH2, ready to use, clone G219-1129, Roche), MutS homolog 6 (MSH6, 1:100, clone 44, Cell Marque, Rocklin, CA, USA), postmeiotic segregation increased 2 (PMS2, 1:40, clone MRQ28, Cell Marque), ERBB2 (ready to use, clone 4B5, Roche), EGFR (1:100, EP38Y, Abcam, Cambridge, UK), c-MET (ready to use, clone SP44, Roche), PTEN (1:100, clone 138G6, Cell Signaling, Danvers, MA, USA), and p53 (1:300, DO7, Novocastra, Newcastle, UK). Epstein-Barr virus-encoded small RNAs (EBER) *in situ* hybridization (ISH) was performed using a Ventana Benchmark ISH system and ISH iView kit (Ventana Medical Systems, Tucson, AZ, USA).

#### *In situ* hybridization for amplification

Gene amplifications identified via OCA and OFA were confirmed using silver ISH (SISH) and fluorescence ISH (FISH). Gene amplification was defined as > 6 gene copies per nucleus or a gene signal/centromere signal ratio > 2.0 according to previous study. [21–27] Probes recognizing the following targets were used: HER2 (INFORM^®^ HER2 DNA and Chr 17 SISH probes, Roche), EGFR (INFORM^®^ EGFR DNA and Chr 7 SISH probes, Roche), MET (INFORM^®^ MET DNA and Chr 7 SISH probes, Roche), FGFR2 (*FGFR2*/CEN10p FISH probe; Abnona Corporation, Taipei, Taiwan), C-myc (*MYC* DNP and Chr 8 probe, Roche), and CCND1 (*CCND1*/CEP11 FISH probe, Vysis, Downers Grove, IL, USA).

### Therapy

Assignment to a matched therapy was determined after reviewing the clinical, laboratory, and pathologic data from all available patient records. Patients whose tumors harbored molecular aberrations were preferably considered for clinical trials with a matched therapy, when available with the following prioritization criteria. (1) Actionable molecular aberrations, wherein any mutation, deletion, or amplification was deemed to be of greater importance; in case with no aberrations, loss of protein of IHC expression was selected. (2) The allocation of patients to investigational treatment varied over time according to the availability of ongoing clinical trials, and off-label treatment, as well as the patient's or physician's preference.

### Statistical analysis

The χ^2^ test was used to assess the correlation between marker status and clinical significance. All the tests were 2-sided, and *P values* of < 0.05 were considered significant. Responses were assessed according to the Response Evaluation Criteria in Solid Tumors (RECIST) criteria version 1.1 [28]. PFS was defined from the first day of treatment to the time of disease progression or death. OS was measured from the time of surgery to death or the last follow-up date and evaluated by survival analysis using the Kaplan-Meier method with a log-rank test. All statistical analyses were performed using SPSS version 18.0 (IBM, Chicago, IL, USA).

## SUPPLEMENTARY MATERIALS, APPENDIX FIGURES AND TABLES




