# Supplementary material for: Complementary utility of targeted next-generation sequencing and immunohistochemistry panels as a screening platform to select targeted therapy for advanced gastric cancer
Source: Oncotarget. 2017 Mar 21;8(24):38389–98. doi: 10.18632/oncotarget.16409 (PMC5503540; doi:10.18632/oncotarget.16409)
Supplement: Supplementary file 1 [file oncotarget-08-38389-s001.pdf]

Complementary utility of targeted next-generation sequencing and immunohistochemistry panels as a screening platform to select targeted therapy for advanced gastric cancer

Supplementary Materials

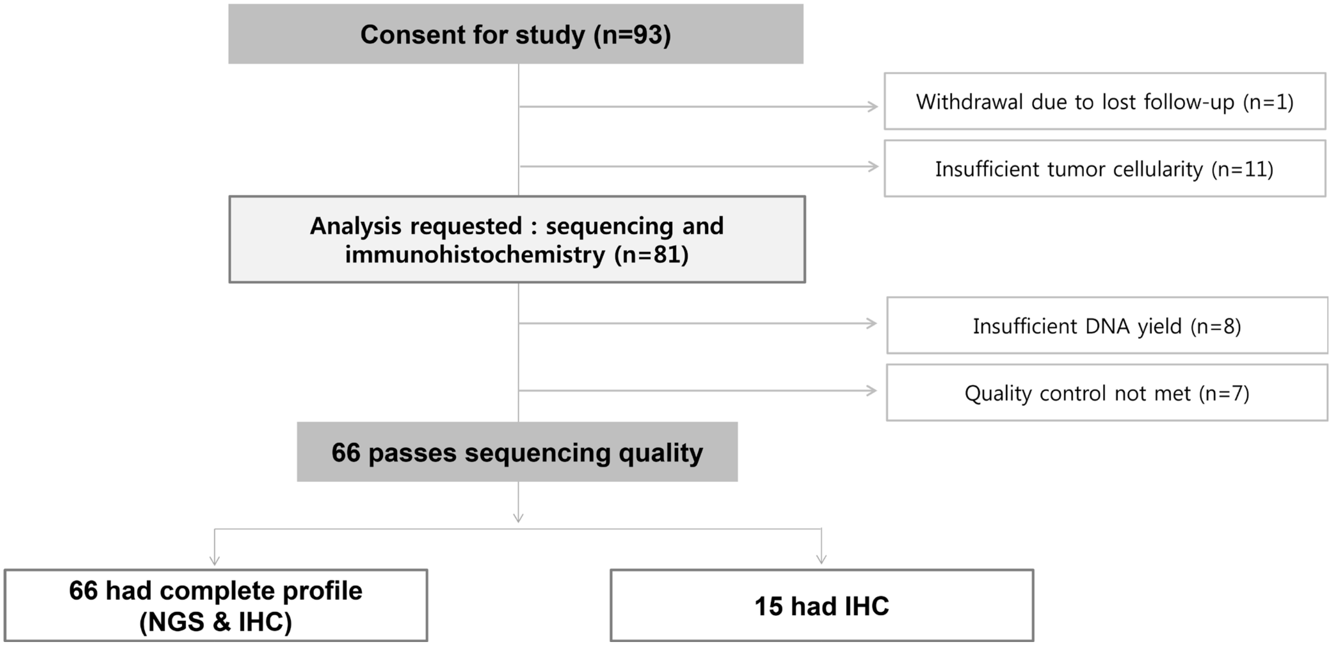

Appendix Figure 1: Study profile.

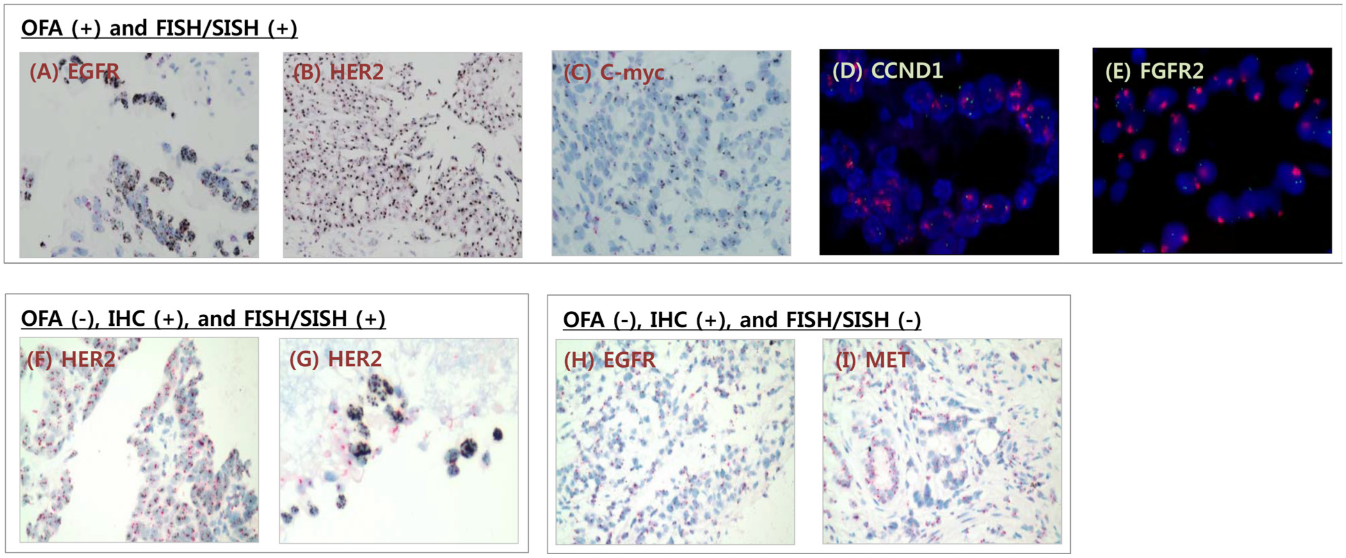

Appendix Figure 2: Representative fluorescence in Situ hybridization (FISH)/silver in situ hybridization (SISH) figures. (**A–E**) Concordant amplified cases between (+) OFA and (+) FISH/SISH. (**F**) and (**G**) Discordant cases; (–) OFA, (+) IHC, and (+) FISH/SISH (**H**) and (**I**) Discordant cases; (–) OFA, (+) IHC, and (–) FISH/SISH.

Appendix Table 1: Gene list and platforms for biomarker analysis (A) Oncomine Focused Assay (B) Oncomine Comprehensive Assay. See Appendix_Table_1

Appendix Table 2: Validation of copy number variations

		OFA		SISH/FISH			IHC	
**Gene**	**Case**	**Clinical Report**	**CNV**	**Copy Number**	**Ratio**	**Result**	**Expression**	**Cellularity**
ERBB2	14	Amplification	150.0	13.6	7.2	Amplified	Positive	50
	15	Amplification	56.7	8.4	4.8	Amplified	Positive	20
	16	Amplification	6.95	4.7	2.9	Amplified	Positive	20
	17	Amplification	33.5	6.5	4.5	Amplified	Positive	40
	32	Amplification	21.7	3.5	1.4	Not-amplified	Negative	50
MYC	18	Amplification	6.9	5.1	2.4	Amplified	-	80
	19	Amplification	8.2	4.7	2.7	Amplified	-	70
	20	Amplification	10.1	9.8	5.9	Amplified	-	50
	21	Amplification	11.7	3.9	2.4	Amplified	-	20
	22	Amplification	12.9	13	11.8	Amplified	-	20
FGFR2	23	Amplification	19.9	16.2	8.7	Amplified	-	80
	24	Amplification	94.8	8.1	3.7	Amplified	-	10
	25	Amplification	21.3	4.3	2.1	Amplified	-	20
CCND1	26	Amplification	10.2	8.0	4.3	Amplified	-	80
	27	Amplification	5.6	8.6	4.8	Amplified	-	30
	28	Amplification	76.6	7.8	3.4	Amplified	-	40
EGFR	29	Amplification	10.5	17.6	8.8	Amplified	Positive	40
	30	Amplification	78.8	11.4	20.4	Amplified	Positive	60

Abbreviations: OFS, Oncomine Focused Assay; FISH, Fluorescence in situ hybridization; IHC, Immunohistochemistry; CNV, copy number variation

Appendix Table 3: Validation of discordant receptor tyrosine kinase (RTK) expressions between oncomine focused assay (OFA) and immunohistochemistry (IHC)

		IHC	SISH/FISH			
**Case**	**Gene**	**Expression**	**Copy number**	**Ratio**	**Result**	**Cellularity (%)**
**OFA (–), IHC (+), and FISH/SISH (+)**						
**33**	**HER2**	3+	5.5	2.4	Amplification	60
**34**	**HER2**	3+	8.3	4.6	Amplification	50
**35**	**EGFR**	3+	18.7	15.2	Amplification	50
**36**	**EGFR**	3+	3.3	0.7	Polysomy*	40
	**MET**	3+	8	3.1	Amplification	
**39**	**MET**	3+	16.8	8.8	Amplification	40
**OFA (–), IHC (+), and FISH/SISH (–)**						
**5**	**EGFR**	3+	2.7	1.5	No amplification	30
	**MET**	3+	2.2	0.9	No amplification	
**11**	**HER2**	3+	3	1.6	No amplification	40
**13**	**EGFR**	3+	3.2	0.78	Polysomy*	20
	**MET**	3+	4.2	1.8	No amplification	
**37**	**EGFR**	3+	1.8	0.4	Polysomy*	10
	**MET**	3+	3.5	0.6	Polysomy*	
**38**	**EGFR**	3+	2.7	1.5	No amplification	60

*Polysomy was defined as CEP 7 ≥4 copies more than 40% of cells (MET) and CEP 7 ≥ 3 copies (EGFR).

Appendix Table 4: Response rate according to matched or non-matched therapy (*n* = 79)

	**Partial response (confirmed)**	**Stable disease**	**Progressive disease**	***P* = 0.002**
Matched	10 (55.6%)	7 (38.9%)	1 (5.6%)	18
Non-matched ramucirumab	5 (16.1%)	19 (61.3%)	7 (22.6%)	31
Non-matched-chemotherapy	3 (10.0%)	24 (80.0%)	3 (10.0%)	30
Total	18 (22.8%)	50 (63.3%)	11 (13.9%)	79
