# Supplementary material for: Complementary utility of targeted next-generation sequencing and immunohistochemistry panels as a screening platform to select targeted therapy for advanced gastric cancer
Source: Oncotarget. 2017 Mar 21;8(24):38389–98. doi: 10.18632/oncotarget.16409 (PMC5503540; doi:10.18632/oncotarget.16409)
Supplement: Supplementary file 2 [file oncotarget-08-38389-s002.docx]

Table A1: Gene list and platforms for biomarker analysis

A) Oncomine Focused Assay

| **Gene** | **Chromosome** | **Amplicon Inclusion** | **Number of Amplicons** |
| --- | --- | --- | --- |
| AKT1 | chr14 | hotspot | 1 |
| ALK | chr2 | hotspot/CNV | 74 |
| AR | chrX | hotspot/CNV | 11 |
| BRAF | chr7 | hotspot/CNV | 25 |
| CCND1 | chr11 | CNV | 10 |
| CDK4 | chr12 | hotspot | 10 |
| CDK6 | chr7 | CNV | 10 |
| CTNNB1 | chr3 | hotspot | 1 |
| DDR2 | chr1 | hotspot | 1 |
| EGFR | chr7 | hotspot/CNV | 11 |
| ERBB2 | chr17 | hotspot/CNV | 11 |
| ERBB3 | chr12 | hotspot | 5 |
| ERBB4 | chr2 | hotspot | 1 |
| ESR1 | chr6 | hotspot | 1 |
| FGFR1 | chr8 | CNV | 14 |
| FGFR2 | chr10 | hotspot/CNV | 20 |
| FGFR3 | chr4 | hotspot/CNV | 36 |
| FGFR4 | chr5 | CNV | 10 |
| GNA11 | chr19 | hotspot | 2 |
| GNAQ | chr9 | hotspot | 2 |
| HRAS | chr11 | hotspot | 2 |
| IDH1 | chr2 | hotspot | 1 |
| IDH2 | chr15 | hotspot | 1 |
| JAK1 | chr1 | hotspot | 3 |
| JAK2 | chr9 | hotspot | 1 |
| JAK3 | chr19 | hotspot | 3 |
| KIT | chr4 | hotspot/CNV | 10 |
| KRAS | chr12 | hotspot/CNV | 10 |
| MAP2K1 | chr15 | hotspot | 3 |
| MAP2K2 | chr19 | hotspot | 1 |
| MET | chr7 | hotspot/CNV | 20 |
| MTOR | chr1 | hotspot | 7 |
| MYC | chr8 | CNV | 10 |
| MYCN | chr2 | CNV | 10 |
| NRAS | chr1 | hotspot | 3 |
| PDGFRA | chr4 | hotspot/CNV | 11 |
| PIK3CA | chr3 | hotspot/CNV | 10 |
| RAF1 | chr3 | hotspot | 5 |
| RET | chr10 | hotspot | 48 |
| ROS1 | chr6 | hotspot | 39 |
| SMO | chr7 | hotspot | 4 |

B) Oncomine Comprehensive Assay

| **Gene** | **Chromosome** | **Amplicon Inclusion** | **Number of Amplicons** |
| --- | --- | --- | --- |
| ABL1 | chr9 | hotspot | 8 |
| ACVRL1 | chr12 | CNV | 17 |
| AKT1 | chr14 | Hotspot/CNV | 18 |
| ALK | chr2 | hotspot | 5 |
| APEX1 | chr14 | CNV | 15 |
| AR | chrX | hotspot/CNV | 16 |
| ARAF | chrX | hotspot | 1 |
| ATM | chr11 | CDS | 157 |
| ATP11B | chr3 | CDS | 15 |
| APC | chr5 | CDS | 117 |
| BAP1 | chr3 | CDS | 34 |
| BCL2L1 | chr20 | CNV | 18 |
| BCL9 | chr1 | CNV | 19 |
| BIRC2 | chr11 | CNV | 16 |
| BIRC3 | chr11 | CNV | 15 |
| BRCA1 | chr17 | CDS | 91 |
| BRCA2 | chr13 | CDS | 137 |
| BRAF | chr7 | hotspot | 3 |
| BTK | chrX | hotspot | 1 |
| CBL | chr11 | hotspot | 3 |
| CCND1 | chr11 | CNV | 19 |
| CCNE1 | chr19 | CNV | 16 |
| CD274 | chr9 | CNV | 18 |
| CD44 | chr11 | CNV | 19 |
| CDH1 | chr16 | CDS | 39 |
| CDKN2A | chr9 | CDS | 12 |
| CDK4 | chr12 | Hotspot/CNV | 15 |
| CDK6 | chr7 | CNV | 15 |
| CHEK2 | cr22 | hotspot | 2 |
| CSF1R | chr5 | hotspot | 3 |
| CSNK2A1 | chr20 | CNV | 19 |
| CTNNB1 | chr3 | hotspot | 13 |
| DCUN1D1 | chr3 | CNV | 14 |
| DDR2 | chr1 | hotspot | 8 |
| DNMT3A | chr2 | hotspot | 5 |
| EGFR | chr7 | hotspot/CNV | 33 |
| ERBB2 | chr17 | hotspot/CNV | 27 |
| ERBB3 | chr12 | hotspot | 2 |
| ERBB4 | chr2 | hotspot | 11 |
| ESR1 | chr6 | hotspot | 3 |
| EZH2 | chr7 | hotspot | 2 |
| FBXW7 | chr4 | CDS | 39 |
| FGFR1 | chr8 | hotspot/CNV | 21 |
| FGFR2 | chr10 | hotspot/CNV | 22 |
| FGFR3 | chr4 | hotspot/CNV | 20 |
| FGFR4 | chr5 | CNV | 19 |
| FLT3 | chr13 | hotspot/CNV | 24 |
| FOXL2 | chr3 | hotspot | 1 |
| GAS6 | chr13 | CNV | 22 |
| GATA2 | chr3 | hotspot | 2 |
| GATA3 | chr10 | CDS | 17 |
| GNA11 | chr19 | hotspot | 1 |
| GNAQ | chr9 | hotspot | 2 |
| GNAS | chr20 | hotspot | 2 |
| HNF1A | chr12 | hotspot | 2 |
| HRAS | chr11 | hotspot | 2 |
| IDH1 | chr2 | hotspot | 3 |
| IDH2 | chr15 | hotspot | 1 |
| IFITM3 | chr11 | hotspot | 2 |
| IGF1R | chr15 | CNV | 19 |
| IL6 | chr7 | CNV | 17 |
| JAK1 | chr1 | hotspot | 2 |
| JAK2 | chr9 | hotspot | 5 |
| JAK3 | chr19 | hotspot | 5 |
| KDR | chr4 | hotspot | 13 |
| KIT | chr4 | hotspot/CNV | 31 |
| KNSTRN | chr15 | hotspot | 1 |
| KRAS | chr12 | hotspot/CNV | 14 |
| MAGOH | chr1 | hotspot | 1 |
| MAPK1 | chr22 | hotspot | 1 |
| MAP2K1 | chr15 | hotspot | 3 |
| MAP2K2 | chr19 | hotspot | 1 |
| MAX | chr14 | hotspot | 2 |
| MCL1 | chr1 | CNV | 17 |
| MDM2 | chr12 | CNV | 14 |
| MDM4 | chr1 | CNV | 20 |
| MED12 | chrX | hotspot | 3 |
| MET | chr7 | hotspot/CNV | 25 |
| MLH1 | chr3 | hotspot | 1 |
| MTOR | chr1 | hotspot | 4 |
| MYC | chr8 | CNV | 11 |
| MYCL | chr1 | CNV | 18 |
| MYD88 | chr3 | hotspot | 3 |
| MPL | chr1 | hotspot | 1 |
| MSH2 | chr2 | CDS | 46 |
| MYCN | chr2 | CNV | 20 |
| MYO18A | chr17 | CNV | 16 |
| NF1 | chr17 | CDS | 138 |
| NF2 | cr22 | CDS | 38 |
| NFEL2 | chr2 | hotspot | 2 |
| NKX2 | chr14 | CNV | 10 |
| NOTCH1 | chr9 | CDS | 103 |
| NPM1 | chr5 | hotspot | 1 |
| NRAS | chr1 | hotspot | 4 |
| PAX5 | chr9 | hotspot | 1 |
| PDCD1LG2 | chr9 | CNV | 17 |
| PDGFRA | chr4 | hotspot/CNV | 22 |
| PIK3CA | chr3 | hotspot/CNV | 36 |
| PIK3R1 | chr5 | CDS | 39 |
| PNP | chr14 | CNV | 18 |
| PPARG | chr3 | CNV | 17 |
| PPP2R1A | chr19 | hotspot | 2 |
| PTCH1 | chr9 | CDS | 67 |
| PTEN | chr10 | CDS | 20 |
| PTPN11 | chr12 | hotspot | 3 |
| RAC1 | chr7 | hotspot | 1 |
| RAF1 | chr3 | hotspot | 1 |
| RB1 | chr13 | CDS | 54 |
| RET | chr10 | hotspot | 5 |
| RHEB | chr7 | hotspot | 1 |
| RHOA | chr3 | hotspot | 1 |
| RPS6KB1 | chr17 | CNV | 18 |
| SF3B1 | chr2 | hotspot | 4 |
| SMAD4 | chr18 | CDS | 9 |
| SMARCB1 | chr22 | CDS | 20 |
| SMO | chr7 | hotspot | 6 |
| SOX2 | chr3 | CNV | 9 |
| SPOP | chr17 | hotspot | 1 |
| SRC | chr20 | hotspot | 2 |
| STAT3 | chr17 | hotspot | 1 |
| STK11 | chr19 | CDS | 22 |
| TET2 | chr4 | CDS | 71 |
| TERT | chr5 | CNV | 15 |
| TIAF1 | chr17 | CNV | 6 |
| TP53 | chr17 | CDS | 24 |
| TSC1 | chr9 | CDS | 57 |
| TSC2 | chr16 | CDS | 94 |
| U2AF1 | chr21 | hotspot | 2 |
| VHL | chr3 | CDS | 8 |
| WT1 | chr11 | CDS | 24 |
| XPO1 | chr2 | hotspot | 1 |
| ZNF217 | chr20 | CNV | 18 |
